# Comparison of three tannases cloned from closely related *lactobacillus* species: *L. Plantarum*, *L. Paraplantarum*, and *L. Pentosus*

**DOI:** 10.1186/1471-2180-14-87

**Published:** 2014-04-07

**Authors:** Shuhei Ueda, Ryohei Nomoto, Ken-ichi Yoshida, Ro Osawa

**Affiliations:** 1 Department of Bioresource Science, Graduate School of Agricultural Science, Kobe University, Rokko-dai 1-1, Nada-ku, Kobe 657-8501, Japan; 2 Department of Agrobioscience, Graduate School Agricultural Science, Kobe University, Rokko-dai 1-1, Nada-ku, Kobe 657-8501, Japan; 3 Research Center for Food Safety and Security, Graduate School Agricultural Science, Kobe University, Rokko-dai 1-1, Nada-ku, Kobe 657-8501, Japan

## Abstract

**Background:**

Tannase (tannin acyl hydrolase, EC 3.1.1.20) specifically catalyzes the hydrolysis of the galloyl ester bonds in hydrolyzable tannins to release gallic acid. The enzyme was found not only in fungal species but also many bacterial species including *Lactobacillus plantarum*, *L. paraplantarum*, and *L. pentosus*. Recently, we identified and expressed a tannase gene of *L. plantarum*, *tanLpl*, to show remarkable differences to characterized fungal tannases. However, little is known about genes responsible for tannase activities of *L. paraplantarum* and *L. pentosus*. We here identify the tannase genes (i.e. *tanLpa* and *tanLpe*) of the above lactobacilli species, and describe their molecular diversity among the strains as well as enzymological difference between species inclusive of *L. plantarum*.

**Results:**

The genes encoding tannase, designated *tanLpa* and *tanLpe*, were cloned from *Lactobacillus paraplantarum* NSO120 and *Lactobacillus pentosus* 21A-3, which shared 88% and 72% amino acid identity with TanLpl, cloned from *Lactobacillus plantarum* ATCC 14917^T^, respectively. These three enzymes could comprise a novel tannase subfamily of independent lineage, because no other tannases in the databases share significant sequence similarity with them. Each of *tanLpl*, *tanLpa*, and *tanLpe* was expressed in *Bacillus subtilis* RIK 1285 and recombinant enzymes were secreted and purified. The K_m_ values of the enzymes on each galloyl ester were comparable; however, the k_cat_/K_m_ values of TanLpa for EGCg, ECg, Cg, and GCg were markedly higher than those for TanLpl and TanLpe. Their enzymological properties were compared to reveal differences at least in substrate specificity.

**Conclusion:**

Two tannase genes responsible for tannase activities of *L. paraplantarum* and *L. pentosus* were identified and characterized. TanLpl, TanLpa and TanLpe forming a phylogenetic cluster in the known bacterial tannase genes and had a limited diversity in each other. Their enzymological properties were compared to reveal differences at least in substrate specificity. This is the first comparative study of closely related bacterial tannases.

## Background

Tannase (tannin acyl hydrolase, EC 3.1.1.20) specifically catalyzes the hydrolysis of the galloyl ester bonds in hydrolyzable tannins that occur widely in the plant kingdom and are considered to be a protective strategy against microbial attack [1]. The enzyme was first reported in fungal genera (e.g. *Aspergillus*, *Penicillium*, and *Candida*[1]) and is used in tea, wine, and beer processing for removal of insoluble condensation products composed of caffeine and tea flavonoids, including catechins [2]. The first indication of bacterial tannase was reported more than 20 years ago, based on methylgallate-hydrolytic activity observed in *Streptococcus gallolyticus* and *Lonepinella koalarum* found in the alimentary tract of koalas feeding on tannin-rich eucalyptus leaves, implying a possible symbiotic relationship between the animal and these bacteria [3-5].

To date, tannase production has been identified in other bacterial species [1], including lactobacilli species of *Lactobacillus plantarum*, *Lactobacillus paraplantarum*, and *Lactobacillus pentosus*, which were isolated from fermented vegetables [6,7]. *L. plantarum*, *L. paraplantarum*, and *L. pentosus* have been reported to play an important role in the production of many fermented foods including dairy products, silage, and pickled vegetables, thus being proposed as a potential probiotic. Furthermore, it has been speculated that their tannase activities in the human alimentary tract have significant effects on pharmacological aspects of dietary tannins that are prevalent in beverages and teas [8]. Recently, we identified *tanLpl* (=lp_2956) within the genome of *L. plantarum* WCFS1 and found it to be similar to a known bacterial tannase gene in *Staphylococcus lugdunensis*[9]. Subsequently, *tanLpl* was expressed in *E. coli*[9,10] to show remarkable differences to characterized fungal tannases. However, little is known about genes responsible for tannase activities of *L. paraplantarum* and *L. pentosus*.

In this study, we aim to identify tannase genes in other lactobacilli, such as *L. paraplantarum* and *L. pentosus* and to compare them with *tanLpl* in *L. plantarum* as well as to distinguish their structural and enzymological characteristics.

## Methods

### Bacterial strains, plasmids, and media

Bacterial strains used in the study and their respective sources are listed in supplementary Additional file 1: Table S1. A total of 27 tannase-producing closely related *Lactobacillus* species isolates, consisting of 8 isolates of *L. plantarum*, 9 isolates of *L. paraplantarum*, 10 isolates of *L. pentosus* were used to study the identification of their tannase encoding genes and the determination of those sequences. The taxonomic identity of *L. plantarum*, *L. paraplantarum*, and *L. pentosus* were determined by a 16S/23S rRNA gene spacer region targeted PCR assay [6]. Among them, *L. plantarum* ATCC 14917^T^, *L. paraplantarum* NOS120 and *L. pentosus* 21A-3 were used as DNA donor strains for the gene cloning and expression of each of the tannases. *Escherichia coli* HST08 (TaKaRa Bio Inc., Shiga, Japan) strain was employed for recombinant plasmid construction. *Bacillus subtilis* RIK 1285 (TaKaRa) was used as the host for gene expression and enzyme purification. The pGEM^®^-T Easy cloning vector (Promega, Madison, USA) and pBE-S vector (TaKaRa) were utilized for the gene cloning for DNA sequencing and heterologous expression of tannase encoding genes in *B. subtilis*, respectively. The lactobacilli strains were cultured statically at 37°C in MRS broth (Difco, Detroit, USA) or on MRS supplemented with 1.5% agar before the experiment.

### Chemicals

Methyl gallate (MG), epicatechin gallate (ECg) epigallocatechin gallate (EGCg) catechin gallate (Cg), and gallocatechin gallate (GCg), used as substrates for enzyme assay of tannases, were obtained from Wako Pure Chemical Industries., Ltd. (Osaka, Japan). In addition, (−)-epigallocatechin-3-*O*–(3-*O*–methyl) gallate (EGCg3″Me) was purchased from Nagara Science (Gifu, Japan). Structures of substrates are shown in Additional file 1: Figure S1. All substrates were dissolved in 50 mM phosphate buffer (pH 6.8) containing 1% ascorbic acid (Wako) at a final concentration of 0.5 mM or 1 mM.

### DNA manipulation

Genomic DNA from the bacterial strains was prepared following the method of Marmur [11]. The purity and amount of DNA in each preparation was estimated spectrophotometrically, and stored at 4°C until use. Isolated genomic DNA was subjected to PCRs along with various sets of primers for cloning of the tannase encoding genes.

All PCR reactions were performed with Ex *Taq* polymerase (TaKaRa). Nucleotide sequences were determined using a BigDye Terminator v3.1 cycle sequencing kit (Applied Biosystems, Warrington, UK) according to the manufacturer’s instructions. Sequencing products were read on an ABI Prism 3100 genetic analyzer (Applied Biosystems, Darmstadt, Germany). The universal primers, T7 promoter primer, and SP6 promoter primer, were used for sequencing.

### Identification of tannase encoding genes of *L. paraplantarum* and *L. pentosus*

Based on the *tanLpl* sequence (GenBank accession no. AB379685), primer pair tanlp-1f (5′-GATTTTTGATGCTGACTGGCT-3′) and tanlp-1r (5′-TAGGCCATGTCTGCGTGTTC-3′) were designed to obtain partial tannase genes in *L. paraplantarum* NSO120 (*tanLpa*) and *L. pentosus* 22A-1 (*tanLpe*). Amplified products were cloned into pGEM-T Easy cloning vector and sequenced. To determine the entire sequences of ORF, inverse PCR was performed as described by Willis et al. [12]. In brief, genomic DNA (1 μg each) of *L. paraplantarum* NSO120 and *L. pentosus* 22A-1 was digested with HincII and SmaI respectively, and purified with the High Pure PCR purification Kit (Roche Diagnostics, Mannheim, Germany) following the manufacturer’s protocol. The recovered DNA were incubated for 12 h at 15°C with 5 U of T4 DNA ligase (TaKaRa) to obtain circularized DNA as templates for inverse PCR using the following primer sets: P1 (5′-AACACGCAGACATGGCCTA-3′) and P2 (5′- ACTTAACGTAACGGATTGCCG-3′) for *tanLpa*, P3 (5′-AAAACTTTAGGAGCCGCCC-3′) and P4 (5′-GCCCGTCCAGCTGAATTTGT-3′) for *tanLpe*. The sequencing of inverse PCR products was performed as described above, and the sequences determined were compared with the tannase gene sequences available in GenBank using the BLAST program (http://blast.ncbi.nlm.nih.gov/Blast.cgi).

### Sequencing of tannase genes in other lactobacilli isolates and their phylogenetic analysis

We designed primers sets based on *tanLpl*, *tanLpa*, and *tanLpe* sequences and following pairs of primers were used to amplify tannase gene sequences in other 24 lactobacilli isolates: tanlpl-F (5′-ATCATTGGCACAAGCCATCA-3′) and tanlpl-R (5′-GGTCACAAGATGAGTAACCG-3′), tanlpa-F (5′-GGTCACAAGATGAGTAACCG-3′) and tanlpa-R (5′-ATTATTGACACAAGTGATCG-3′), and tanlpe-F (5′-ATGACGGATGCTTTGATTTT-3′) and tanlpe-R (5′-CTACTGACACAGGCCATCGA-3′). The amplified PCR fragment was cloned into pGEM-T Easy cloning vector, and the DNA sequence was determined.

The deduced amino acid sequences of TanLpl, TanLpa, and TanLpe were aligned by the ClustalW method using the MEGA5 software package [13]. Phylogenetic trees were constructed using the neighbor-joining method [14] with MEGA5. The percentage of similarity between nucleotide sequences was calculated using BioEdit software [15]. The average ratios of non-synonymous and synonymous substitutions (dN/dS) were estimated using the modified Nei–Gojobori/Jukes–Cantor method in MEGA5.

### Construction of *tanLpl*, *tanLpa*, and *tanLpe* expression plasmids

The coding regions of *tanLpl*, *tanLpa*, and *tanLpe* were amplified by PCR using the following three primers pairs: for TanLpl; 5′-CAT*ATG*TAACCGATTGCTTTTTGATG-3′ (start codon of *tanLpl* is shown in italics and an NdeI site is underlined) and 5′-AAGCTTTTGGCACAAGCCATCAATCCAGGA-3′ (HindIII site is underlined), for TanLpa; 5′-CAT*ATG*AGTAACCGATTGATTTTTGATG-3′ (start codon of *tanLpa* is shown in italics and an NdeI site is underlined) and 5′-AAGCTTTTGACACAAGTGATCAATCCAGGC-3′ (HindIII site is underlined), and for TanLpe; 5′-CAT*ATG*ACGGATACTTTGATTTTTGATG-3′ (start codon of *tanLpe* is shown in italics and an NdeI site is underlined) and 5′-GGATCCCTGACACAGGCCATCGATCCA-3′ (BamHI site is underlined). The amplified fragment was cloned into pGEM-T Easy cloning vector and sequenced to confirm the absence of PCR errors. The plasmid was digested with NdeI and HindIII, or NdeI and BamHI, and the resultant 1.4-kb DNA fragment was ligated with pBE-S vector (TaKaRa) that had been digested with NdeI and HindIII, or NdeI and BamHI, to generate pBE-*tanLpl*, -*tanLpa*, and -*tanLpe*. Those of pBE-S construct in which the ORF for tannase genes were fused with sequences for 173 unique signal peptides and the ligated products were transformed into *B. subtilis* RIK 1285 using *B. subtilis* Secretory Protein Expression System (TaKaRa) according to manufacture’s protocol. The transformed cells were plated onto LB agar plates containing 50 μg/ml ampicillin and 30 μg/ml kanamycin. To screen for the clones with high tannase activity, the spectrophotometric method of Sharma et al. [16] was used.

### Enzyme purification

For the production of TanLpl, TanLpa, and TanLpe that contained His tags at the C-termini, the transformed *B. subtilis* RIK 1285 cells that showed highest tannase activities were inoculated into LB medium (200 ml) containing 50 μg/ml ampicillin and 30 μg/ml kanamycin and grown for 24 h at 37°C with gentle shaking. Cells were harvested by centrifugation at 10,000 × *g* for 5 min and resuspended in buffer A (50 mM Tris–HCl pH 8.0, 500 mM NaCl, 10 mM imidazole, and 10% glycerol) containing 1 mg/ml lysozyme and then disrupted by rigorous shaking with glass beads (ϕ0.1 mm) for 5 min. The crude cell-free extract was prepared by removal of cell debris by centrifugation at 10,000 × *g* for 20 min. The recombinant protein with a His tag was purified using a TALON® Metal Affinity Resin (TaKaRa) according to the manufacturer’s instructions, with the exception that 10% glycerol was added to all buffers. The purified recombinant tannase proteins were dialyzed against buffer B (10 mM Tris–HCl pH 8.0, 50 mM NaCl, and 20% glycerol). The purity of the recombinant proteins was checked by SDS-polyacrylamide gel electrophoresis. The protein concentration was measured with a NanoDrop 2000 spectrophotometer (Thermo Scientific, Wilmington, DE).

### Amino acid sequencing

The N-terminal amino acid sequence of TanLpl, TanLpa, and TanLpe were determined by automated Edman degradation using a PPSQ-10 protein sequencer (Shimadzu, Kyoto, Japan).

### Effects of pH and temperature on tannase activity

The activity of the purified recombinant TanLpl, TanLpa, and TanLpe on pH and temperature was determined in comparison with that of a commercially available *A. oryzae* tannase (Wako). All reaction mixtures contained 600 nM of the purified tannase and 1 mM MG as a substrate. The optimal pH of the enzyme was determined at 37°C for 15 min in the range of pH 4.0–10.0 using the following buffers: 50 mM sodium citrate buffer (pH 4.0–5.5), 50 mM phosphate buffer (pH 6.0–7.0), 50 mM Tris–HCl buffer (pH 7.5–8.5), and 50 mM NaHCO_3_ buffer (pH 9.0–10.0). The optimum temperature was determined by measuring the tannase activity at 20–55°C in 50 mM Tris–HCl (pH 8.0) for TanLpl, TanLpa, and TanLpe, and in 50 mM sodium citrate (pH 5.5) for *A. oryzae* tannase. The reaction products were analyzed by high performance liquid chromatography (HPLC) as described previously [17]. One unit of tannase activity was defined as the amount of enzyme required to release 1 μmol of gallic acid in 1 min under specified conditions.

### Effects of various chemicals on tannase activity

Effects of various metal ions (CaCl_2_, MnCl_2_, FeSO_4_, MgSO_4_, ZnSO_4_), EDTA, urea, β-mercaptoethanol, and phenylmethylsulfonyl fluoride (PMSF) on the lactobacilli tannase activities were investigated. Activity of each enzyme was estimated using 1 mM MG as substrate with 1 mM each of the above chemicals at 37°C for 15 min under the predetermined optimal pH condition. The reaction products were analyzed by HPLC as described above.

### Kinetic constant of Lactobacilli tannase

The reaction mixture (200 μl) was prepared in 50 mM Tris–HCl (pH 8.0) for TanLpl, TanLpa, and TanLpe, or 50 mM sodium citrate (pH 5.5) for *A. oryzae* tannase, containing each of the substrates (0.1–4 mM), and the enzyme (33 nM). The mixture without enzyme was once preincubated at 37°C for 10 min, and the reaction was started by adding the enzyme. After incubation at 37°C for 15 min, the reaction was stopped by adding 20 μl of 20% (v/v) phosphoric acid to be subjected directly to HPLC analysis. *K*_m_ and *V*_max_ values were calculated from a Hanes–Woolf plot. *k*_cat_ value was calculated based on the molecular mass of each tannase enzyme (deduced from the gene sequences and SDS-PAGE).

### Nucleotide Sequence Accession Number

The nucleotide sequences reported in this study has been submitted to DDBJ/EMBL/GenBank under the accession number listed in Additional file 1: Table S1.

## Results

### Sequence analysis of *tanLpl*, t*anLpa*, and *tanLpe*

The full-length nucleotide sequence of the *tanLpa* (1410 bp) of *L. paraplantarum* NSO120 and *tanLpe* (1413 bp) of *L. pentosus* 22A-1 as determined by inverse PCR predicted proteins of 469 and 470 amino acid residues, with molecular mass of 50,708 Da and 51,193 Da, respectively. TanLpa and TanLpe turned out to be homologs of TanLpl with 88% and 72% amino acid identity, respectively. The average dN/dS ratios for three lactobacilli tannase was 0.1373 suggesting that these genes are under neutral (dN/dS = 1) or purifying selection (dN/dS < 1). The levels of sequence identity to other known bacterial tannases, such as TanA from *S. lugdunensis* and two putative tannase-coding genes from the whole genome sequence of *S. gallolyticus* UCN34 (GenBank accession no. YP_003430356 and YP_003431024) were less than 30% (Additional file 1: Figure S2). However, alignment analysis revealed that these enzymes contained a highly conserved Gly-X-Ser-X-Gly motif (e.g. the 161th to 165th positions of TanLpl sequence), typical of the catalytic triad with a nucleophilic serine found in serine hydrolases [18] (Additional file 1: Figure S2). Although the enzymes were supposed to be secreted, SignalP 4.1 server (http://www.cbs.dtu.dk/services/SignalP/) analysis failed to suggest any plausible signal peptide sequence.

**Figure 1:**
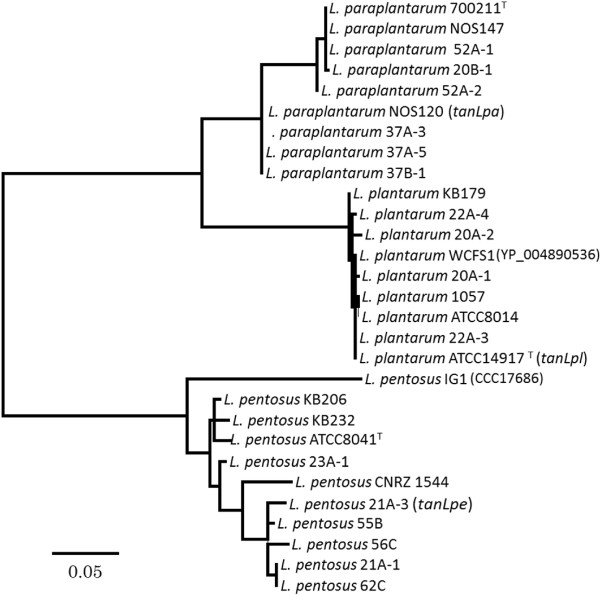
**Neighbor-joining phylogenetic consensus tree based on amino acid sequences of TanLpl, TanLpa, and TanLpe.** The deduced amino acid sequences of TanLpl, TanLpa, and TanLpe were aligned by the ClustalW method using the MEGA5 software package [12]. Phylogenetic trees were constructed using the neighbor-joining method [13] with MEGA5. The percentage of similarity between nucleotide sequences was calculated using BioEdit software [14]. The analysis was based on 469 residues for TanLpl and TanLpa sequences, and 470 residues for TanLpe sequences. The tannase genes of the *L. plantarum* WCFS1 (GenBank accession no. YP_004890536) and *L. pentosus* IG1 (GenBank accession no. CCC17686) were used to align with the corresponding genes obtained in this study. The stability of the groupings was estimated by bootstrap analysis with 1,000 replications. The information of used strains and DDBJ accession numbers are listed in Additional file 1: Table S1.

We sequenced the tannase-coding genes from 24 additional isolates of *L. plantarum*, *L. paraplantarum*, and *L. pentosus* (Additional file 1: Table S1). Their amino acid sequences composed the clades subdividing the species ranged from 99.3%-100% for *L. plantarum*, 95.5%-100% for *L. paraplantarum*, and 93.8%-100% for *L. pentosus* (Figure 1). The comparative analysis revealed that the lactobacilli tannase genes had a restricted diversity, forming a distinct phylogenetic cluster among the known tannases (Additional file 1: Figure S3). TanLpl, TanLpa, and TanLpe are representing a novel subfamily as they showed low amino acid sequence similarity less than 60% with any other reported tannases in DDBJ/EMBL/GenBank databases.

### Expression and purification of recombinant tannase

**Figure 2:**
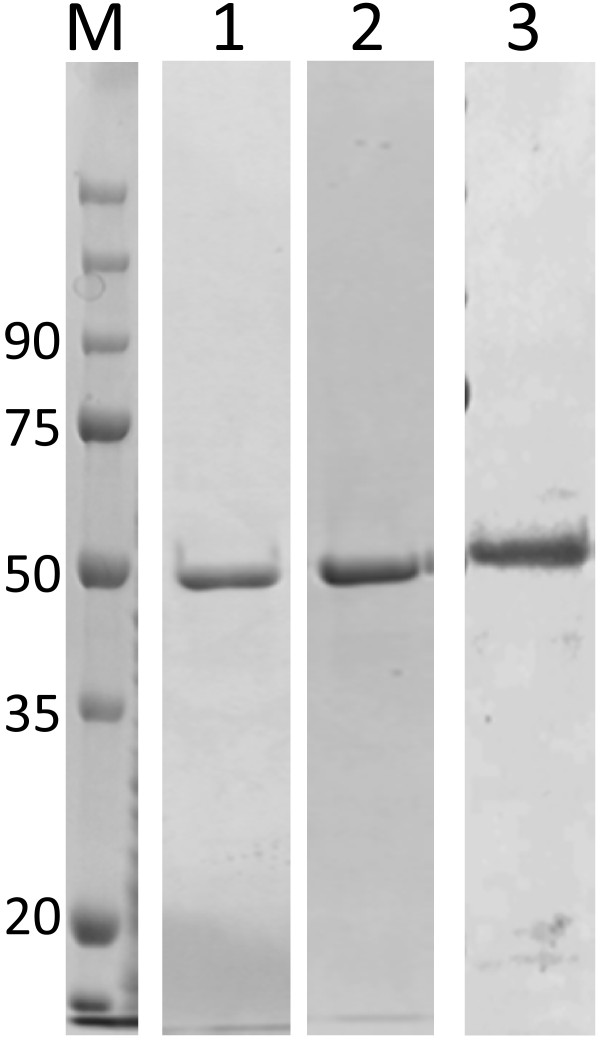
**Purification of the recombinant tannase proteins.** Proteins were examined by 10%. SDS–PAGE. Lane M, protein molecular-weight markers (labelled in kDa); lane 1, purified TanLpl; lane 2, purified TanLpa; lane 3, purified TanLpe. All recombinant proteins were purified by TALON resin column.

It should be noted that we did not obtain any clone that secreted a measurable amount of recombinant tannase protein in the spent medium. Therefore, we obtained the purified recombinant enzymes from bacterial cells of the clones of transformed *B. subtilis*. Each of the three lactobacilli tannase genes (i.e. *tanLpl*, *tanLpa*, and *tanLpe*) was expressed as C-terminal His-tag fusion proteins with N-terminal secretion signal peptide which were originating from YbdK protein, which was selected from several clones showed high tannase activity, under the control of *aprE* promoter in *B. subtilis* RIK 1285. In all cases, no tannase activity was found in the culture media, while washed *B. subtilis* cells showed appreciable activity. Moreover, only after tannase activity appeared in the supernatant of cultures the lysozyme treatment providing the protoplast, suggesting that the secreted recombinant tannases might be associated with the cell wall. The cells (ca. 1.5 g [wet weight]) were harvested and disrupted by shaking with glass beads prior to purification of the recombinant tannases were purified by the metal affinity chromatography to the purities greater than 95% (Figure 2). Molecular masses of the recombinant TanLpl, TanLpa, and TanLpe were approximately 50 kDa, 50 kDa, and 51 kDa, respectively (Figure 2), which well agreed with the estimation from their respective amino acid sequences. Amino acid sequencing confirmed that the N-terminal sequences of purified TanLpl, TanLpa, and TanLpe matched the corresponding sequence predicted from *tanLpl*, *tanlpa*, and *tanlpe*, respectively.

### Effects of pH, temperature, and chemicals on tannase activity

**Figure 3:**
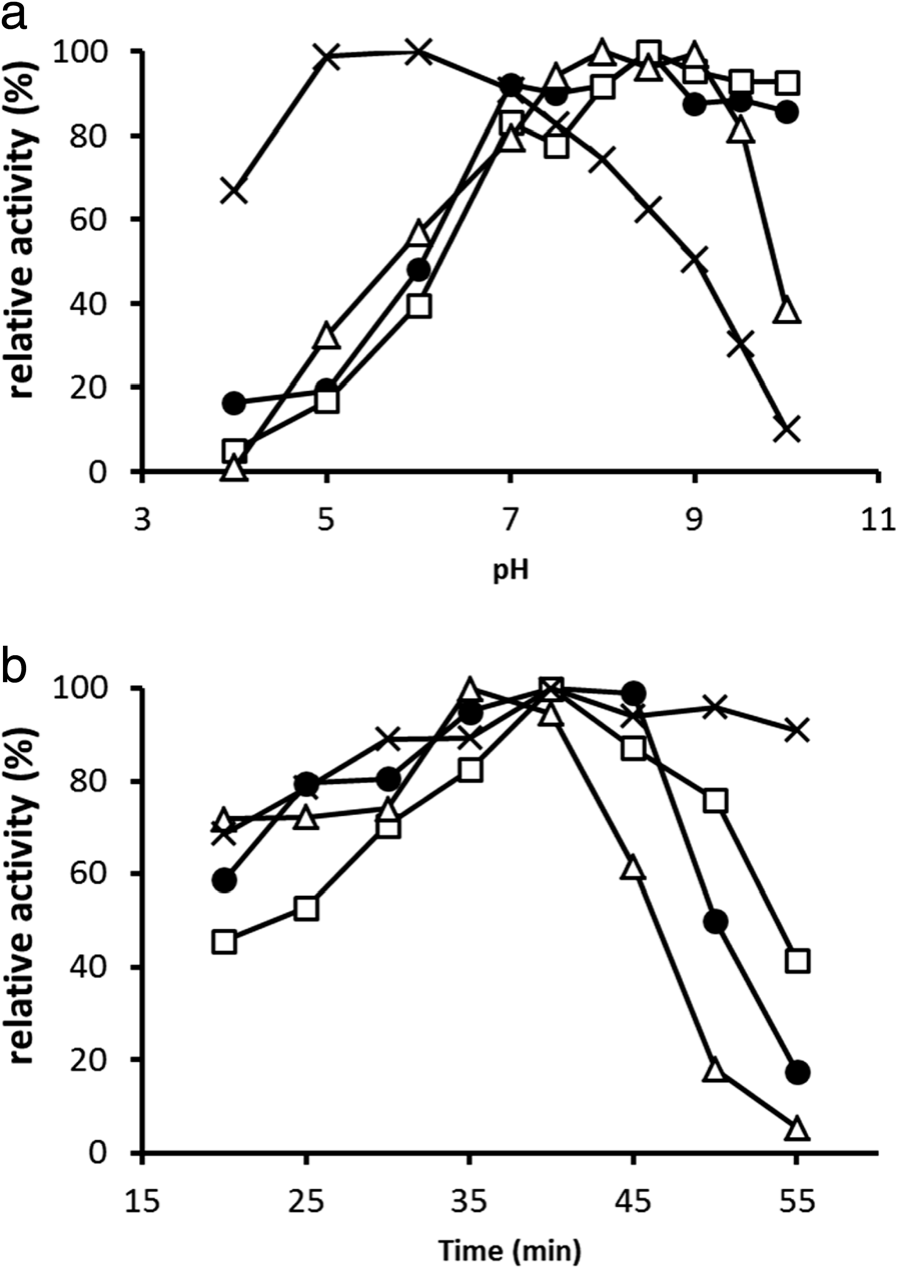
**Effects of pH (a) and temperature (b) on the activities of TanLpl (•), TanLpa (□), TanLpe (△), and *A. oryzae *tannase (×).** HPLC analysis was performed under various conditions for the hydrolysis of methyl gallate. pH experiments were performed at 37°C, and temperature experiments were performed at pH 8.0 and 5.5 for lactobacilli tannase and *A. oryzae* tannase, respectively. The values are shown as the relative activity, and the maximum relative activities are indicated as 100%. Each experiment was performed in triplicate.

Enzymatic properties of the lactobacilli tannases were investigated using MG as a substrate. TanLpl and TanLpa showed maximum activities at pH 8.5 and at 40°C, whereas those of TanLpe were optimal at pH 8.0 and at 35°C (Figure 3a, b). Although TanLpl and TanLpa sustained more than 80% of their enzymatic activities at a pH range of 8.0–10.0, TanLpe drastically lost its activity above pH 9.0. In addition, the activity of TanLpe was always lower than that of TanLpl and TanLpa at temperatures higher than 40°C. In contrast, the activity of *A. oryzae* tannase showed a maximum level at approximately pH 5.5 and 45–50°C, while it dropped drastically at pH values above 5.5 and below 4.5, but retained more than 50% activity was between 20°C and 60°C (Figure 3a, b).

The tannase activities of the purified recombinant enzymes were partially inhibited by but not significantly affected by PMSF, EDTA, and urea (Table 1). Some metal ions including Mn^2+^, Ca^2+^, and Mg^2+^ slightly interfered the enzymes. Especially, TanLpl and TanLpe were affected to decrease the activity down to 46.1% and 25.2% by the presence of Zn^2+^, respectively. All three recombinant tannases were inhibited in the presence of 1 mM FeSO_4_ approximately down to one fourth levels (Table 1).

**Table 1:** **Effects of various chemicals on relative activities of recombinant tannases**^**a**^

	**Rel act. (%)**		
**Chemicals (1 mM)**	**TanLpl**	**TanLpa**	**TanLpe**
Control	100	100	100
MnCl_2_	87.6 ± 22.5	111.3 ± 23.8	75.6 ± 13.2
CaCl_2_	98.3 ± 15.8	88.7 ± 11.5	92.3 ± 12.7
FeSO_4_	22.5 ± 12.2	24.1 ± 18.4	23.4 ± 13.1
ZnSO_4_	46.1 ± 7.64	95.4 ± 16.3	25.2 ± 17.5
MgSO_4_	123.7 ± 20.1	110.5 ± 11.9	96.7 ± 7.0
PMSF	83.2 ± 14.7	66.2 ± 20.3	81.2 ± 24.7
EDTA	97.6 ± 3.0	87.8 ± 4.2	103.7 ± 12.2
Urea	91.4 ± 8.8	96.9 ± 0.37	119.5 ± 18.3

^a^Assays were carried out in triplicate and the results represent the means ± standard deviations.

### Kinetic properties of TanLpl, TanLpa, and TanLpe

*K*_m_ values of TanLpl, TanLpa, and TanLpe for the other catechin derivatives were approximately 10 times lower than those for MG (Table 2). *k*_cat_/*K*_m_ values of TanLpa for catechin derivatives, except for EGCg3″Me, were markedly higher than those of not only TanLpl and TanLpe (Table 2) but also *A. oryzae* tannase (Additional file 1: Table S2). *k*_cat_/*K*_m_ values of these three enzymes for EGCg3″Me were the lower of all the tested substrates.

**Table 2:** **Kinetic properties of TanLpl, TanLpa, and TanLpe**^**a**^

**Substarate**	**TanLpl**			**TanLpa**			**TanLpe**		
	***K***_**m **_**(mM)**	***k***_***c*** **at**_**(s**^**-1**^**)**	***k***_**cat**_**/*K***_**m **_**(s**^**-1**^ **· mM**^**-1**^**)**	***K***_**m **_**(mM)**	***k***_***c*** **at**_**(s**^**-1**^**)**	***k***_**cat**_**/*K***_**m **_**(s**^**-1**^ **· mM**^**-1**^**)**	***K***_**m **_**(mM)**	***k***_***c*** **at**_**(s**^**-1**^**)**	***k***_**cat**_**/*K***_**m **_**(s**^**-1**^ **· mM**^**-1**^**)**
Methyl gallate (MG)	0.37 ± 0.04	46.02 ± 0.87	125.02 ± 15.43	0.50 ± 0.06	72.73 ± 3.34	145.12 ± 10.65	0.87 ± 0.41	15.95 ± 3.13	18.79 ± 3.08
Epicatechin gallate (ECg)	0.03 ± 0.02	1.49 ± 0.19	52.23 ± 25.64	0.06 ± 0.01	11.08 ± 0.44	195.30 ± 21.53	0.05 ± 0.01	0.42 ± 0.03	8.63 ± 1.17
Epigallocatechin gallate (EGCg)	0.10 ± 0.01	1.12 ± 0.03	11.68 ± 1.29	0.06 ± 0.01	14.29 ± 0.82	260.76 ± 46.52	0.06 ± 0.02	0.44 ± 0.02	7.25 ± 2.51
Catechin gallate (Cg)	0.05 ± 0.002	2.41 ± 0.10	53.65 ± 4.62	0.05 ± 0.005	8.1 ± 0.04	181.5 ± 27.71	0.08 ± 0.004	1.48 ± 0.11	19.22 ± 2.36
Gallocatechin gallate (GCg)	0.03 ± 0.008	0.89 ± 0.044	27.19 ± 6.28	0.06 ± 0.002	9.2 ± 0.09	154.68 ± 7.97	0.07 ± 0.002	1.12 ± 0.13	14.32 ± 1.95
Epigallocatechin-3-*O*-(3-*O*-methyl) gallate (EGCg3″Me)	0.04 ± 0.009	0.26 ± 0.04	6.04 ± 0.57	0.04 ± 0.004	0.35 ± 0.07	9.02 ± 2.28	0.005 ± 0.0009	0.06 ± 0.02	10.57 ± 1.33

^a^Assays were carried out in triplicate and the results represent the means ± standard deviations.

## Discussion

In this study, *tanLpa* from *L. paraplantarum* NSO120 and *tanLpe* from *L. pentosus* 22A-1, cloned to reveal their high amino acid identity to TanLpl from *L. plantarum*. Recently, Ren et al. [19] reported a crystal structure of tannase from *L. plantarum*, that has 99% amino acid identity to TanLpl. They identified Ser163, His451, and Asp419 as a catalytic triad with a nucleophilic serine within the pentapeptide sequence motif Gly161-X-Ser163-X-Gly165 of the crystal structure. Alignment analysis indicated that all the three lactobacilli tannases, TanLpl, TanLpa, and TanLpe contained the conserved Gly-X-Ser-X-Gly motif in their amino acid sequences as the catalytic triad (Additional file 1: Figure S1). In addition, we found that amino acid residues of Asp421, Lys343, and Glu357, considered to play a key role in binding of the enzyme to them corresponding galloyl site of the substrate [19], were also conserved.

We sequenced a total of 28 possible lactobacilli tannase genes, forming a distinct phylogenetic clade among the tannase genes reported in databases. No other bacterial tannases in databases showed higher than 60% amino acid sequence similarity with TanLpl, TanLpa, or TanLpe, suggesting that the three lactobacilli tannases form a novel independent lineage of tannase superfamily.

Although an increasing number of genome sequencing reports are revealing that bacteria possess various tannase genes, only few of them have been cloned and expressed in heterologous hosts [20]. We thus undertook the gene expression and protein purification of TanLpl, TanLpa, and TanLpe in *B. subtilis*. However, the recombinant tannases were not readily secreted into the culture medium, but were trapped within the cell walls. In agreement with our previous report [9], the optimum temperature and pH for activities of TanLpl were 40°C and 8.5, respectively. On the other hand, Rodríguez et al. [21] reported that cell-free extracts of the type strain *L. plantarum* CECT 748^T^ (=ATCC 14917^T^) had optimal tannase activity at pH 5.0 and at 30°C. According to the available genome information of *L. plantarum* ATCC 14917^T^, this strain is known to have at least two unique tannase genes in its genome, i.e., *tanLpl* and another gene (GenBank accession no. ZP_07077992). It might be possible that Rodríguez et al. [21] worked with the second one. The optimum temperature and pH of TanLpa were similar to those of TanLpl, whereas TanLpe was weaker at temperatures higher than 40°C. The number of proline residues was reported to contribute to the enzyme thermo-stability [22]. The difference might be due to the lower proline content of TanLpe (21 proline residues), compared with TanLpl (23 proline residues) and Tanlpa (25 proline residues).

Most of lactobacilli species are acid tolerant reflecting the fact that they produce various organic acids during fermentation, and thought until recently, to be generally not considered alkali sensitive. Nevertheless, Sawatari et al. [23] reported that some lactobacilli strains including *L. plantarum* and *L. pentosu*s originating from plant materials showed growth at pH up to 8.9 and alkali tolerance of the glycolytic enzymes of the strains. Moreover, in turned out that *L. pentosus* CECT 5138 has been used as a starter to ferment lye-treated green olives at pH higher than 9.0 [24]. These facts and findings suggest that some lactobacilli are able to tolerate a very alkaline environment in some occasions for their survival. Nishitani et al. [7] speculated that tannase production might allow *L. plantarum* strains to accumulate high intracellular levels of Mn^2+^, which is otherwise chelated by tannins, compensating for the absence of superoxide dismutase in *L. plantarum* and providing resistance to oxygen toxicity under aerobic conditions. If this is actually the case, the alkaline tolerant tannases may be beneficial for their survival under alkaline conditions.

It was reported that tannase activities were affected by several chemicals [10,25]. The activities of recombinant TanLpl, TanLpa, and TanLpe were significantly affected by neither EDTA, Mn^2+^, Mg^2+^, Ca^2+^, PMSF, nor urea. Previously Curiel et al. [10] reported that the activity of TanLpl was greatly increased in the presence of Ca^2+^ and was significantly inhibited in the presence of urea. Notably, they used *E. coli* as a host for the expression of a recombinant N-terminal His-tagged TanLpl while we used *B. subtilis* and C-terminal His-tagged recombinant. In order to clarify the inconsistency further characterization is required, but the effects of EDTA, Mn^2+^, and Mg^2+^ were in good agreement.

TanLpl, TanLpa, and TanLpe were not affected in 1 mM EDTA, implying that the enzymes do not depend on divalent metallic ions as co-factors. Only the exception was Fe^2+^, which was also shown to inhibit a tannase from *Penicillium chrysogenum*[26].

Previously [9]*K*_m_ value of TanLpl for MG was found to be lower than that of commercially available tannase of *A. orzae*. In this study, *K*_m_ and *k*_cat_ values of TanLpl, TanLpa, and TanLpe were calculated not only for MG, but also for various catechin galloyl esters. *K*_m_ values of the enzymes on each these substrates were comparable; however, *k*_cat_/*K*_m_ values of TanLpa for EGCg, ECg, Cg, and GCg were significantly higher than those of TanLpl and TanLpe. The results suggest that the small differences in the amino acid sequences of tannase can exert such a drastic effect. Interestingly, *k*_cat_/*K*_m_ values for EGCg3″Me of TanLpl and TanLpa were much lower than those for other catechins (Table 2). EGCg3″Me is a derivative of EGCg, in which the methoxyl group is located at the galloyl group of EGCg. Ren et al. [19] showed that the hydrogen-bonding network which was observed between three hydroxyl groups of gallic acid and the side chains of Asp421, Lys343, and Glu357 of lactobacilli tannase is important in enzyme-substrate complex. Therefore, EGCg3″Me may not form a strong and stable complex with the enzymes.

Tannins are widely distributed in the plant kingdom and bind readily with proteins or heavy metals to form insoluble complexes, thereby presumably acting as a defense mechanism in plants against microbial attacks [7,27]. Tannins, such as tannic acid, methyl gallate, and catechin galloyl ester derivatives are structurally diverse. Our study suggests that variety in bacterial tannases may reflect adaptation to various tannin substrates present in the environment. This is the first comparative study of closely related bacterial tannases, which may be as functionally diverse as bacterial β-glucosidases required for the break down of the plant-based glucosides [28], reflecting the possible “co-evolutional arms race” between plants and bacteria.

## Conclusion

In the present study, we identified the genes encoding tannase, designated *tanLpa* and *tanLpe*, were cloned from *Lactobacillus paraplantarum* NSO120 and *Lactobacillus pentosus* 21A-3, which shared 88% and 72% amino acid identity with TanLpl, cloned from *Lactobacillus plantarum* ATCC 14917^T^, respectively. Our comparative analysis showed that *Lactobacillus* tannase genes had a little diversity in each other, forming a phylogenetic cluster in the known tannase genes *in silico*. Meanwhile, TanLpl, TanLpa, and TanLpe that were recombinant enzymes of *tanLpl*, *tanLpa*, and *tanLpe* expressed in *Bacillus subtilis* RIK 1285 showed appreciable difference in enzymological acitivity against several galloyl esters, in which TanLpa, for example, had markedly higher catalytic activity than TanLpl and TanLpe against some galloyl esters of green tea catechins (i.e. epigallocatechin gallate, epicatechin gallate, catechin gallate, gallocatechin gallate). This is the first comparative study of closely related bacterial tannases.

## Competing interests

The authors declare that they have no competing interests.

## Authors’ contributions

SU carried out the molecular genetic studies and enzymatic analysis, participated in the sequence alignment, purification the recombinant enzymes, and kinetic analysis. RN performed the data analysis, participated in the design of the study, and drafted the manuscript. KY helped to draft the manuscript. RO conceived of the study, and participated in its design and helped to draft the manuscript. All authors read and approved the final manuscript.

## Supplementary Material

Additional file 1: Table S1 The strains used in this study. **Table S2.** Kinetic properties of *A. orazae* tannase. **Figure S1.** Chemical structures of substrates used in this study. MG: methyl gallate, Cg: catechin gallate, GCg: gallocatechin gallate, ECg: epicatechin gallate, EGCg: epigallocatechin, gallate, EGCg3″Me: (-)-epigallocatechin-3-*O*-(3-*O*-methyl) gallate. **Figure S2.** Alignment of bacterial tannases. The sequences of TanA (*Staphylococcus ludunensis*),*S. gallolyticus* tannase 1 (*Streptococcus gallolyticus*, accession no. YP_003430356), and *S. gallolyticus* tannase 2 (accession no. YP_003431024) were obtained from the Genbank database. G-X-S-X-G motif is indicated with red color bar. **Figure S3.** Phylogenetic tree analysis of tannase superfamily homologous to TanLpl, TanLpa, and TanLpe by Maximum. Likelihood Method. Total of 22 predicted bacterial tannase proteins were selected for the phylogenetic tree analysis. Click here for file
