# Supplementary material for: Comparison of three tannases cloned from closely related lactobacillus species: L. Plantarum, L. Paraplantarum, and L. Pentosus
Source: BMC Microbiol. 2014 Apr 7;14:87. doi: 10.1186/1471-2180-14-87 (PMC4233993; doi:10.1186/1471-2180-14-87)
Supplement: Additional file 1: Table S1 — The strains used in this study. Table S2. Kinetic properties of A. orazae tannase. Figure S1. Chemical structures of substrates used in this study. MG: methyl gallate, Cg: catechin gallate, GCg: gallocatechin gallate, ECg: epicatechin gallate, EGCg: epigallocatechin, gallate, EGCg3″Me: (-)-epigallocatechin-3-O-(3-O-methyl) gallate. Figure S2. Alignment of bacterial tannases. The sequences of TanA (Staphylococcus ludunensis),S. gallolyticus tannase 1 (Streptococcus gallolyticus, accession no. YP_003430356), and S. gallolyticus tannase 2 (accession no. YP_003431024) were obtained from the Genbank database. G-X-S-X-G motif is indicated with red color bar. Figure S3. Phylogenetic tree analysis of tannase superfamily homologous to TanLpl, TanLpa, and TanLpe by Maximum. Likelihood Method. Total of 22 predicted bacterial tannase proteins were selected for the phylogenetic tree analysis. [file 1471-2180-14-87-S1.pdf]

Table S1. The strains used in this study

Strain no.	Species	Isolated from	Source	GenBank accession no. of tannase gene
ATCC14917^T	*L. plantarum*	Pickled cabbage	ATCC	AB379685
JCM1057	*L. plantarum*	Corn silage	JCM	AB794905
ATCC8014	*L. plantarum*	unknown	ATCC	AB794909
20A-1	*L. plantarum*	Turnip pickled with rice bran	Our collection	AB794906
20A-2	*L. plantarum*	Turnip pickled with rice bran	Our collection	AB794907
22A-3	*L. plantarum*	Eggplant pickled with rice bran	Our collection	AB794908
22A-4	*L. plantarum*	Eggplant pickled with rice bran	Our collection	AB794911
KB179	*L. plantarum*	Human feces	Our collection	AB794910
ATCC700211^T	*L. paraplantarum*	Beer contaminant	ATCC	AB794894
37A-3	*L. paraplantarum*	Takana pickles	Our collection	AB794889
20B-1	*L. paraplantarum*	Turnip pickled with rice bran	Our collection	AB794888
37B-1	*L. paraplantarum*	Takana pickles	Our collection	AB794891
37A-5	*L. paraplantarum*	Takana pickles	Our collection	AB794890
52A-1	*L. paraplantarum*	Turnip pickled with rice bran	Our collection	AB794892
52A-2	*L. paraplantarum*	Turnip pickled with rice bran	Our collection	AB794893
NOS 120	*L. paraplantarum*	Kimchi	Our collection	BAN10246
NOS 147	*L. paraplantarum*	Pickled Chinese cabbage	Our collection	AB794895
ATCC8041^T	*L. pentosus*	Silage	ATCC	AB794901
23A-1	*L. pentosus*	Vegetable pickled with rice bran	Our collection	AB794897
55B	*L. pentosus*	Eggplant pickled with rice bran	Our collection	AB794898
56C	*L. pentosus*	Eggplant pickled with rice bran	Our collection	AB794899
62C	*L. pentosus*	Cucumber pickled with rice bran	Our collection	AB794900
KB232	*L. pentosus*	Human feces	Our collection	AB794904
K206	*L. pentosus*	Human feces	Our collection	AB794903
21A-1	*L. pentosus*	Vegetable pickled with rice bran	Our collection	AB794896
21A-3	*L. pentosus*	Vegetable pickled with rice bran	Our collection	BAN10247
CNRZ 1544	*L. pentosus*	Fermented olives	Our collection	AB794902

Table S2. Kinetic properties of *A. oryzae* tannase[a]

Substarate	*A. oryzae* tannase		
	K_m (mM)	k_{cat} (s^{-1})	k_{cat}/K_m ($s^{-1} \cdot mM^{-1}$)
methyl gallate (MG)	3.61 ± 0.18	28.62 ± 0.63	7.97 ± 0.83
epicatechin gallate (ECg)	0.12 ± 0.02	5.27 ± 0.09	43.73 ± 8.22
epigallocatechin gallate (EGCg)	0.15 ± 0.01	7.19 ± 0.15	45.49 ± 21.68
catechin gallate (Cg)	0.16 ± 0.01	7.21 ± 0.20	44.65 ± 1.63
gallocatechin gallate (GCg)	0.28 ± 0.04	18.36 ± 0.2	54.59 ± 17.3
epigallocatechin-3-*O* -	0.06 ± 0.008	5.01 ± 0.2	73.11 ± 5.5

a Assays were carried out in triplicate and the results represent the means ± standard deviations.

FIG S1

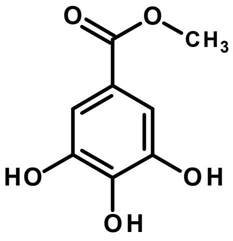

MG

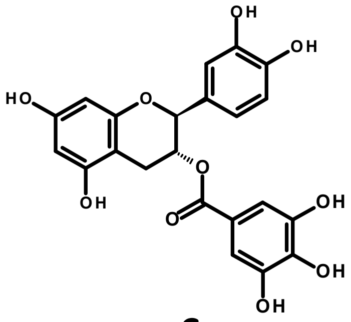

Cg

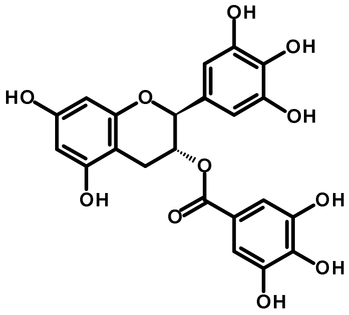

GCg

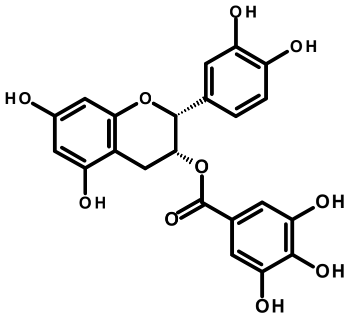

ECg

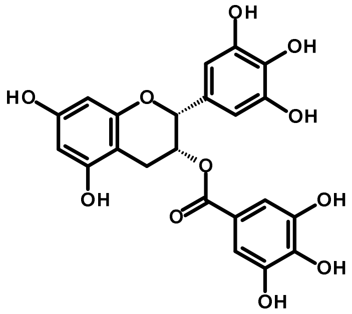

EGCg

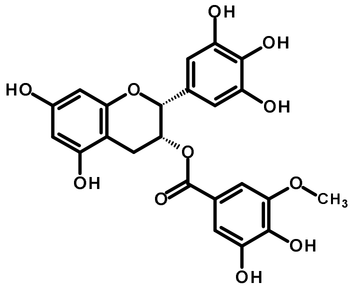

EGCg3″Me

Fig. S1. Chemical structures of substrates used in this study.
MG: methyl gallate, Cg: catechin gallate,GCg: gallocatechin gallate, ECg: epicatechin gallate, EGCg: epigallocatechin gallate, EGCg3″Me: (-)-epigallocatechin-3-*O*-(3-*O*-methyl) gallate.

FIG S2

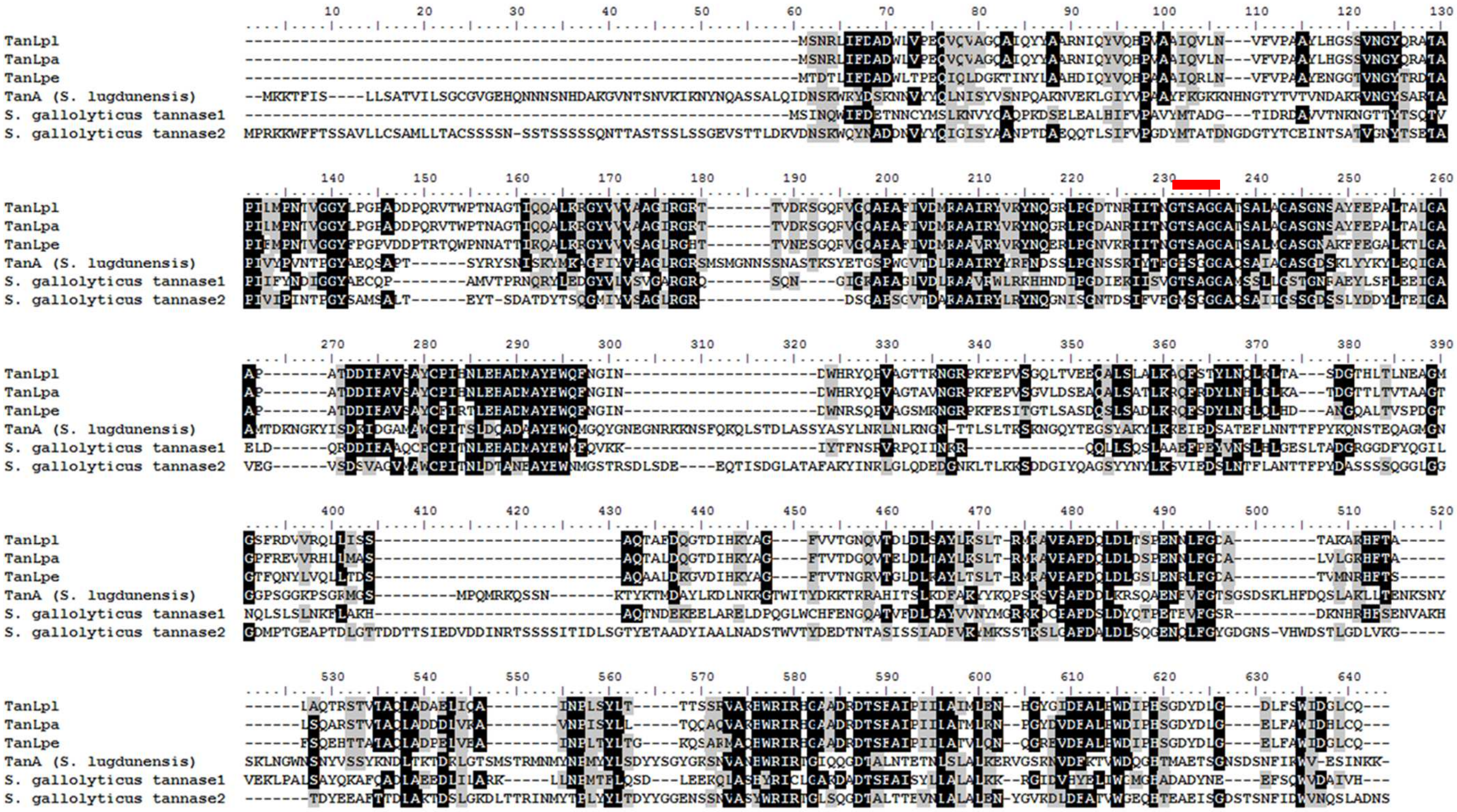

Fig. S2. Alignment of bacterial tannases.
The sequences of TanA (*Staphylococcus ludunensis*),*S. gallolyticus* tannase 1 (*Streptococcus gallolyticus*, accession no. YP_003430356), and *S. gallolyticus* tannase 2 (accession no. YP_003431024) were obtained from the Genbank database. G-X-S-X-G motif is indicated with red color bar.

FIG S3

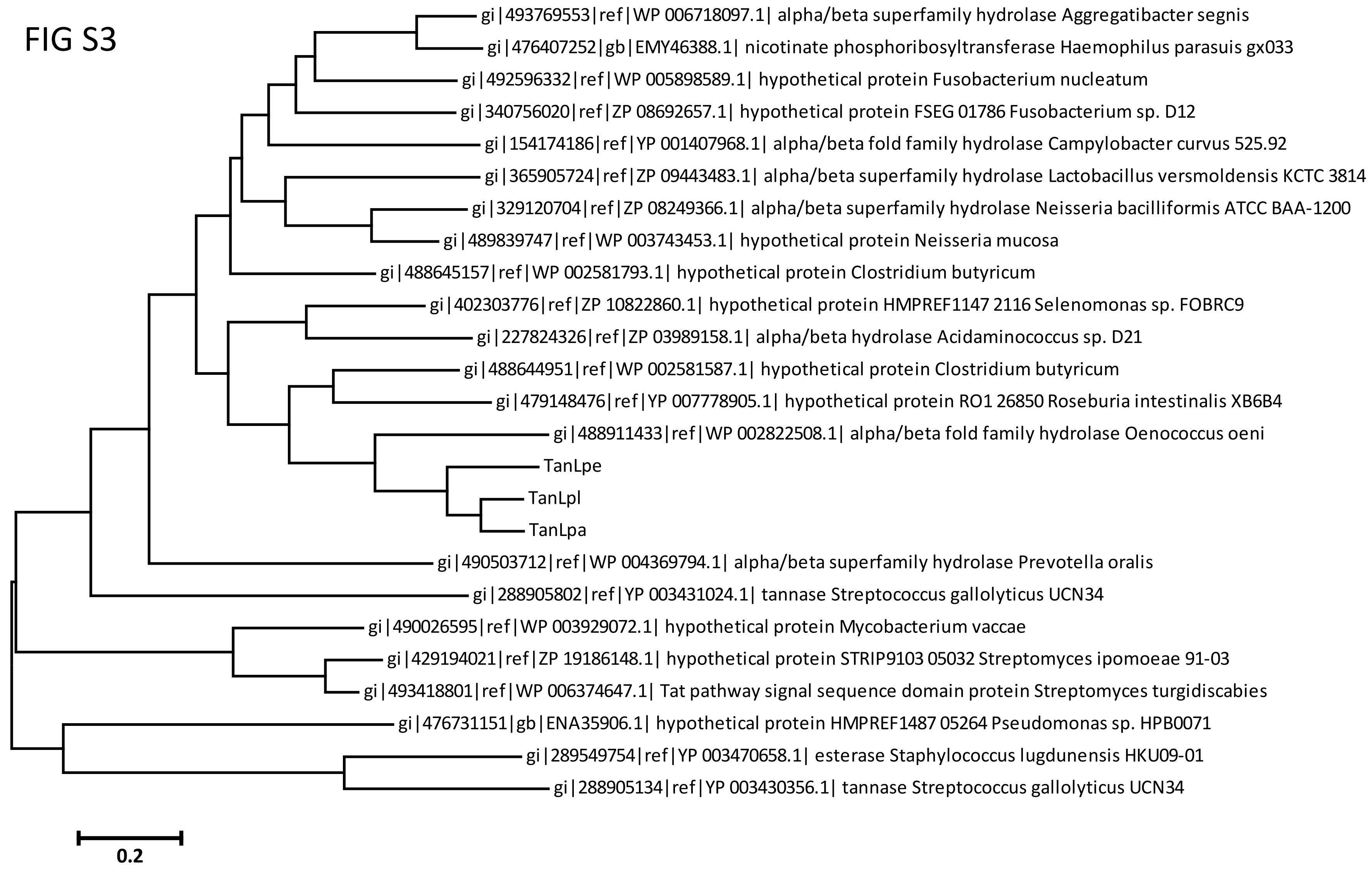

Fig. S3. Phylogenetic tree analysis of tannase superfamily homologous to TanLpl, TanLpa, and TanLpe by Maximum Likelihood Method. Total of 22 predicted bacterial tannase proteins were selected for the phylogenetic tree analysis.
